# Performance, Egg Quality, and Intestinal Morphology of Laying Hens Fed High-Fiber Diets with or Without Stimbiotic Supplementation †

**DOI:** 10.3390/ani16050700

**Published:** 2026-02-24

**Authors:** Amanda Fabrício Dantas de Lima, Ricardo Romão Guerra, Isabelle Naemi Kaneko, Danilo Vargas Gonçalves Vieira, Danilo Teixeira Cavalcante, Matheus Ramalho de Lima, Adiel Vieira de Lima, Paloma Eduarda Lopes de Souza, Carlos Henrique do Nascimento, Edijanio Galdino da Silva, Xavière Rousseau, Fernando Guilherme Perazzo Costa, Germano Augusto Jerônimo do Nascimento

**Affiliations:** 1 Department of Animal Science, Center of Agricultural Sciences, Federal University of Paraiba, Areia 58397-000, Paraiba, Brazil; amandadantas550@gmail.com (A.F.D.d.L.); ricardo@cca.ufpb.br (R.R.G.); adiel1205@hotmail.com (A.V.d.L.); palomaeduardasbu@gmail.com (P.E.L.d.S.); carlosnascimento889@gmail.com (C.H.d.N.); edijanio@veterinario.med.br (E.G.d.S.); 2 Department of Animal Science, Federal University of Rondonia, Campus of Presidente Médici, Presidente Médici 76916-000, Rondonia, Brazil; isabelle.naemi@unir.br; 3 Department of Animal Science, Federal University of Northern Tocantins, Araguaína University Campus, Araguaína 77804-970, Tocantins, Brazil; danilo.vieira@ufnt.edu.br; 4 Department of Animal Science, Federal University of Agreste of Pernambuco, Garanhuns 55292-278, Pernambuco, Brazil; danilo.cavalcante@ufape.edu.br; 5 Department of Animal Science, Federal Rural University of the Semi-Arid Region, Mossoró Campus, Mossoró 59625-900, Rio Grande do Norte, Brazil; mrlmatheus@ufersa.edu.br; 6 Ab Vista, 3 Woodstock Court, Marlborough SN8 4AN, UK; xaviere.rousseau@abvista.com

**Keywords:** egg production, egg quality, fermentable fiber, gut health, xylanase, wheat bran

## Abstract

Moderately fermentable dietary fiber (with different crude fiber [CF] levels), especially when combined with stimbiotic (STB) supplementation, can enhance intestinal health, nutrient utilization, and overall performance in laying hens, although effects depend on fiber type, level, and diet composition. To investigate this, 1200 Bovans White laying hens (32 weeks old) were assigned to a 2 × 6 factorial experiment with two levels of supplementation (without or with 0.01% STB) and six dietary fiber treatments: Control (commercial diet), Corn–soybean, 75:25 wheat–corn, 50:50 wheat–corn, 25:75 wheat–corn, and Corn–soybean. The study spanned five 28-day periods, evaluating productive performance, egg quality, and intestinal morphology. Dietary fiber levels significantly improved feed intake (*p* = 0.0029), egg production (*p* < 0.0001), egg mass (*p* < 0.0001), feed conversion (*p* < 0.0001), and intestinal structure (*p* ≤ 0.05), while STB alone had limited effects. Hens fed 75:25 and 50:50 wheat–corn diets consumed more feed, and the highest egg production and mass were observed in layers receiving Control, 75:25 wheat–corn, and Corn–soybean diets. Egg quality benefited from the fiber–STB interaction, producing heavier eggs with higher yolk pigmentation (*p* ≤ 0.05), thicker shells (*p* ≤ 0.05), and specific gravity (*p* ≤ 0.05). STB supplementation increased jejunal villus width (*p* = 0.0001) and absorptive area (*p* = 0.0063), whereas fiber type affected ileal villus width (*p* = 0.0025) and absorptive area (*p* = 0.0156). Fiber–STB interaction influences the duodenum villus width (*p* = 0.0106), crypt depth (*p* = 0.0011), villus-to-crypt ratio (*p* = 0.0058), and absorptive area (*p* = 0.0086), and ileum villus width (*p* = 0.0011), crypt depth (*p* = 0.0058), and absorptive area (*p* = 0.0086). In conclusion, the use of 0.01% STB in diets with high crude fiber levels (25:75 and 75:25 wheat–corn ratios) improves performance, egg quality, and intestinal health in laying hens.

## Simple Summary

Poultry farming faces the challenge of producing eggs efficiently while ensuring the health and welfare of laying hens. Nutrition is one of the main factors influencing performance, egg quality, and intestinal health. In this study, we evaluated the combined effects of a stimbiotic feed additive and different levels of dietary fiber in laying hens. The results showed that the combination of stimbiotic and higher fiber levels improved layer performance, egg quality, and intestinal morphology. This research provides practical insights for poultry producers seeking more efficient and environmentally responsible strategies to support animal health and food production.

## 1. Introduction

Historically, dietary fiber has been considered a low-value component in poultry nutrition, viewed as an energy diluent and associated with the presence of antinutritional factors [1]. However, recent studies show that its effect is highly dependent on solubility and fermentability, which can positively or negatively impact performance and gut health [2,3]. Moderately fermentable fiber can stimulate gastrointestinal tract development, enhance endogenous enzyme production, and modulate the microbiota, resulting in improved nutrient utilization and immune responses [4,5,6].

In laying hens, this issue is even more relevant due to their prolonged production cycle, increasing animal welfare demands, and the frequent use of fibrous ingredients in commercial diets [7]. Nevertheless, the presence of soluble non-starch polysaccharides (NSPs), especially arabinoxylans, can increase digesta viscosity, impair digestion and microbiota balance, and predispose layers to enteric disorders [8,9,10].

Commercial diets are most commonly based on corn, which contains low levels of arabinoxylans and fiber, limiting the effectiveness of xylanase in enhancing fiber fermentation. Conversely, the inclusion of wheat bran tends to increase the availability of substrates for enzyme activity. Wheat bran is an insoluble fiber source rich in arabinoxylans—estimated at around 23.2% [11]. Studies have shown that moderate inclusion of wheat bran improves intestinal health in poultry by providing energy to enterocytes through fermentation of arabinoxylo-oligosaccharides, producing SCFAs. Walugembe et al. [1] showed increased cecal SCFAs and improved microbiota in broilers and layers aged 1–21 days. Veluri et al. [12] demonstrated that stimbiotic supplementation (β-1,4-xylanase + xylo-oligosaccharides) enhanced growth, villus height, and gut health in broilers, with effects influenced by fiber particle size. Nguyen et al. [13] reported that xylanase improved nutrient utilization, egg quality, and performance in laying hens aged 20–40 weeks. Singh and Kim [14] reviewed that soluble and moderately fermentable fibers, combined with enzymes or prebiotics, positively modulate microbiota, SCFA production, and intestinal morphology. Qiu et al. [15] showed that Bacillus-based probiotics synergize with fermentable fibers to enhance growth, intestinal morphology, and microbial composition in broilers aged 1–42 days.

Although studies in broilers have consistently shown positive effects of STB, results in laying hens remain limited and inconsistent, varying according to fiber type and level, diet composition, and production conditions [2,3,6]. Thus, a knowledge gap remains regarding which dietary fiber levels, with or without STB supplementation, can simultaneously optimize performance, egg quality, and intestinal integrity in commercial layers.

Therefore, this study evaluated the effect of different dietary high-fiber levels, with or without STB supplementation, on productive performance, egg quality, and intestinal morphology of laying hens.

## 2. Materials and Methods

### 2.1. Animal Breeding and Management

This study was conducted at Campus II of the Federal University of Paraíba, located in the city of Areia, in Paraíba, Brazil. All protocols and procedures followed animal welfare guidelines and were approved by the local Ethics Committee of the Federal University of Paraíba (protocol code 5673170325).

A total of 1200 Bovans White laying hens, 32 weeks of age, were used in the study. These layers were obtained at one day of age and managed according to the instructions described in the strain manual until the beginning of the experimental phase. The hens were housed in conventional laying facilities with clay-tiled roofs, equipped with trough feeders and nipple drinkers. They were kept in galvanized wire cages measuring 100 × 45 × 45 cm.

### 2.2. Experimental Diets and Design

The diets were formulated to meet the nutritional requirements of the Bovans White strain, considering an average intake of 120 g/layer/day, according to Rostagno et al. [16]. The experimental design was conducted in a 2 × 6 factorial arrangement, with 10 replicates of 10 layers per replicate (N = 100 layers per treatment), during five periods of 28 days each. The levels of supplementation were (without or with 0.01% stimbiotic—STB) and six dietary fiber levels, consisting of: 1. Control (commercial diet); 2. Corn–soybean; 3. 75:25 wheat–corn; 4. 50:50 wheat–corn; 5. 25:75 wheat–corn; 6. Wheat–soybean; 7. Control (commercial diet) + STB; 8. Corn–soybean + STB; 9. 75:25 wheat–corn + STB; 10. 50:50 wheat–corn + STB; 11. 25:75 wheat–corn + STB; 12 Wheat–soybean + STB (Table 1). The STB (Signis, β-1,4-endo-xylanase and xylo-oligosaccharides, AB Vista, Marlborough, UK) was supplemented at 100 mg/kg of feed, providing an activity of 16,000 BXU/kg. One BXU (xylanase unit) corresponds to the amount of enzyme required to release 1 nmol of reducing sugars from birchwood xylan per second at 50 °C and pH 5.3.

### 2.3. Experimental Variables

#### 2.3.1. Performance

The variables evaluated included feed intake (FI, g/layer/day). To determine FI, the residual feed was weighed and subtracted from the amount of feed initially provided for the entire period. At the end of each 28-day period, the amount of feed consumed was divided by the number of hens in each treatment and by the number of days to calculate the average grams of feed consumed per hen per day.

Egg production (EP, %) was determined by recording the number of eggs produced per day, including broken, cracked, and abnormal eggs (e.g., soft-shelled eggs). This value represented the average EP of the hens during each period.

Egg mass (EM, g) was calculated by multiplying the average egg weight by the total number of eggs produced during the experimental period.

Feed conversion ratio per egg mass (FCR-EM, g/g) was calculated as the total FI divided by the total EM produced (kg/kg). Feed conversion ratio per dozen eggs (FCR-DZ, g/dozen) was calculated as the total FI (kg) divided by the number of dozens of eggs produced.

Body weight variation (BWV, g) was obtained by weighing the hens at the beginning and at the end of the experimental phase. The average BWV is expressed in grams per hen.

#### 2.3.2. Egg Quality

Egg quality analyses were performed during the last three days of each 28-day period. Three eggs with average weight from each replicate (N = 30 eggs per treatment in each evaluation period) were collected, individually identified, and weighed on an analytical balance. Subsequently, the eggs were broken onto a flat surface to measure albumen height (mm) using a depth micrometer (model S-8400, Ames^®^, Boston, MA, USA).

Yolk and albumen weights were then recorded. The shells were dried in a forced-air oven at 45 °C for 48 h and subsequently weighed. Percentages of each component were calculated by dividing the weight of the component by the total egg weight and multiplying by 100.

Yolk color was assessed using the DSM Yolk Color Fan scale (DSM, São Paulo, Brazil).

The Haugh Unit was determined using the equation proposed by [17]: HU = 100 × log (H − 1.7 × W^0.37^ + 7.57), where HU = Haugh Unit, H = albumen height (mm), and W = egg weight (g).

Eggshell thickness was assessed with a digital micrometer at three evenly spaced locations on the equatorial region of the shell, and the arithmetic mean was used as the representative value.

Specific gravity was determined using the saline flotation method. Eggs were immersed in sodium chloride (NaCl) solutions with densities ranging from 1.0700 to 1.0975 g/cm^3^, with a gradient of 0.0025 between successive solutions, according to methodology Hamilton (1982) [18]. The density of the solutions was regularly verified using an oil densimeter.

#### 2.3.3. Intestinal Morphology

At the end of the experiment, one layer per replicate was euthanized to collect biological samples. A 1 cm fragment was obtained from the middle segments of the duodenum, jejunum, and ileum of each bird for each treatment. These fragments were immediately fixed in formalin solution, neutral buffered, 10% (n° HT501850, Darmstadt, Germany).

Following standard histological procedures, the tissue samples were embedded in paraffin. Sections of 5 µm thickness were then cut from each paraffin block using a microtome. The slides were stained with periodic acid–Schiff (PAS) (n° 3952016, Sigma-Aldrich, St. Louis, MO, USA) and examined using a Motic camera (Motic Instruments Inc., Xiamen, China) attached to an Olympus BX-53 microscope (Olympus Corporation, Tokyo, Japan) with Motic Image Plus 2.0 software for image analysis.

For each photomicrograph, three measurements of villus height and crypt depth were taken, resulting in a total of 90 measurements per variable per treatment (10 birds × 3 photomicrographs × 3 measurements). Villus height and width (µm) were measured from the mucosal region corresponding to the upper portion of the crypts up to the villus apex. Crypt depth (µm) was defined as the distance from the villus base to the crypt–villus junction. The villus-to-crypt ratio was calculated as the ratio of villus height to crypt depth. The absorptive surface area (µm^2^) of the villus was estimated by modeling each villus as a cylindrical structure, calculated using the formula [19]:

Villus absorptive surface area = 2π × (average villus width/2) × villus height

### 2.4. Statistical Analysis

Data were analyzed as a 2 × 6 factorial using the PROC GLM procedure of SAS (version 9.4, SAS Institute Inc., Cary, NC, USA). The factors included stimbiotic supplementation (0 or 100 g/ton of feed) and levels of dietary fiber 1. Control (commercial diet); 2. Corn–soybean; 3. 75:25 wheat–corn; 4. 50:50 wheat–corn; 5. 25:75 wheat–corn; 6. Corn–soybean). Significance was set at *p* ≤ 0.05 and tendency was declared at 0.05. Only the results showing significant interactions between the factors are presented. Significantly different means were separated using Tukey’s HSD for fiber level effects, and the F-test was applied to assess the effects of STB.

## 3. Results

### 3.1. Performance

There was no significant interaction between dietary fiber levels and STB supplementation. Independently, STB supplementation did not affect feed intake (FI), egg production (EP), egg mass (EM), feed conversion per egg mass (FCR-EM) or per dozen eggs (FCR-DZ), or body weight variation (BWV). However, dietary fiber levels significantly influenced all variables evaluated (*p* < 0.05; Table 2).

Hens fed the 50:50 wheat–corn and 75:25 wheat–corn diets had higher FI compared with the other treatments (*p* = 0.0029). The highest EP and EM were obtained from hens fed the Control (commercial diet), 75:25 wheat–corn, and Corn–soybean diets (*p* < 0.0001). Consequently, these diets also promoted better FCR-EM (*p* < 0.0001) and FCR-DZ (*p* < 0.0001). Regarding BWV, hens fed the Corn–soybean, 25:75 wheat–corn, and 75:25 wheat–corn diets exhibited less body weight loss at the end of the experimental period (*p* = 0.0152).

### 3.2. Egg Quality

Interactions between STB supplementation and dietary fiber levels were observed for yolk color, shell thickness, and eggshell specific gravity (*p* < 0.0001) (Table 3 and Table 4). The main effect of STB supplementation significantly affected Haugh unit (*p* = 0.0222) and specific gravity (*p* < 0.0001).

Laying hens fed the Control (commercial diet), 25:75 wheat–corn, and 75:25 wheat–corn diets produced heavier eggs (*p* < 0.0001). More intensely pigmented yolks were obtained from hens fed the Control (commercial diet) and Corn–soybean diets (*p* < 0.0001). The highest Haugh unit and specific gravity values were observed in eggs from hens fed the Corn–soybean diet (*p* < 0.0001), whereas thicker eggshells were produced by hens fed the 75:25 wheat–corn diet (*p* = 0.0198). Dietary fiber levels did not influence the percentage of yolk, albumen, or eggshell.

Table 3 details the interactions between dietary fiber levels and STB supplementation. Eggs from hens fed the 25:75 wheat–corn diet supplemented with STB exhibited darker yolk pigmentation, like those from hens receiving the 50:50 wheat–corn diet without STB. The Control (commercial diet) diet supplemented with STB resulted in thicker eggshells (*p* < 0.0001). Moreover, STB supplementation in the Corn–soybean, 50:50 wheat–corn, or 25:75 wheat–corn diets significantly increased specific gravity (*p* < 0.0001).

### 3.3. Intestinal Morphology

**Figure 1:**
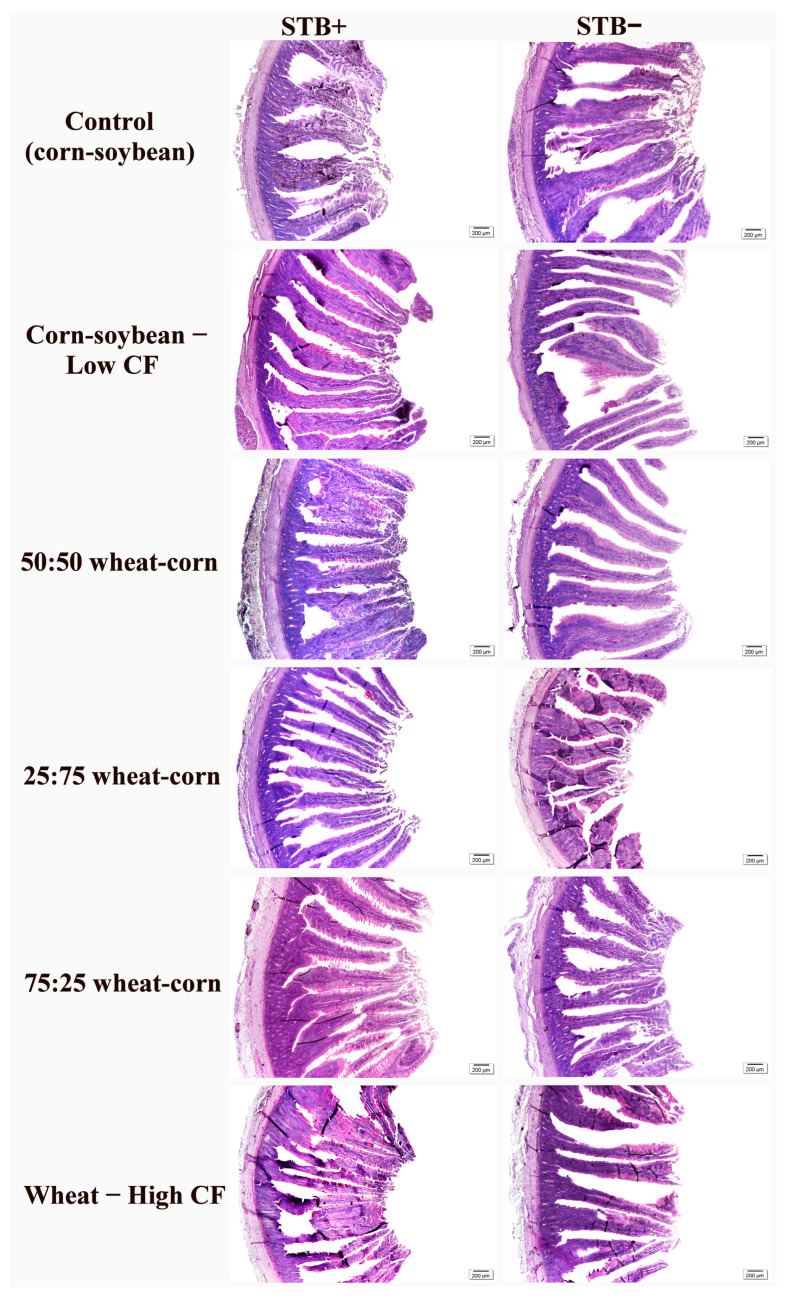
Photomicrographs of the duodenum in laying hens, supplemented with dietary fiber levels and STB supplementation. Control (corn–soybean); Corn–soybean—Low CF; 50:50 wheat–corn; 25:75 wheat–corn; 75:25 wheat–corn e Wheat—High CF. Staining: PAS. Scale bars: 200 µm.

**Figure 2:**
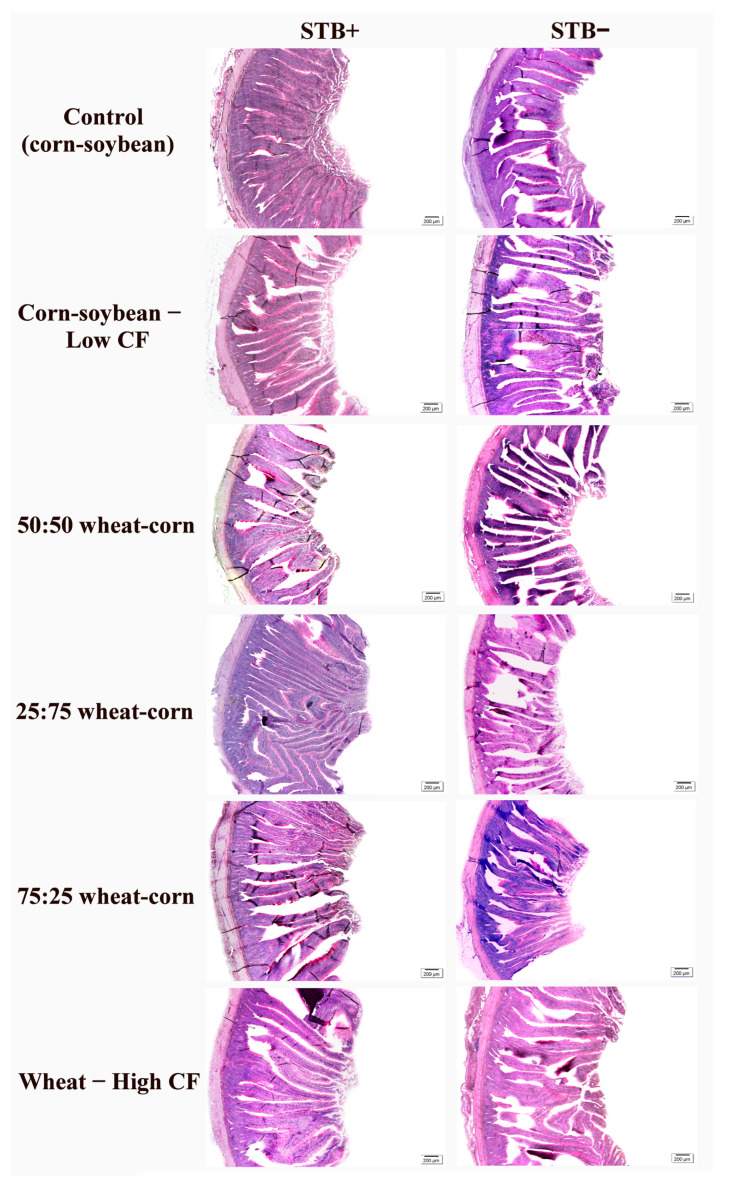
Photomicrographs of the jejunum in laying hens, supplemented with dietary fiber levels and STB supplementation. Control (corn–soybean); Corn–soybean—Low CF; 50:50 wheat–corn; 25:75 wheat–corn; 75:25 wheat–corn e Wheat—High CF. Staining: PAS. Scale bars: 200 µm.

**Figure 3:**
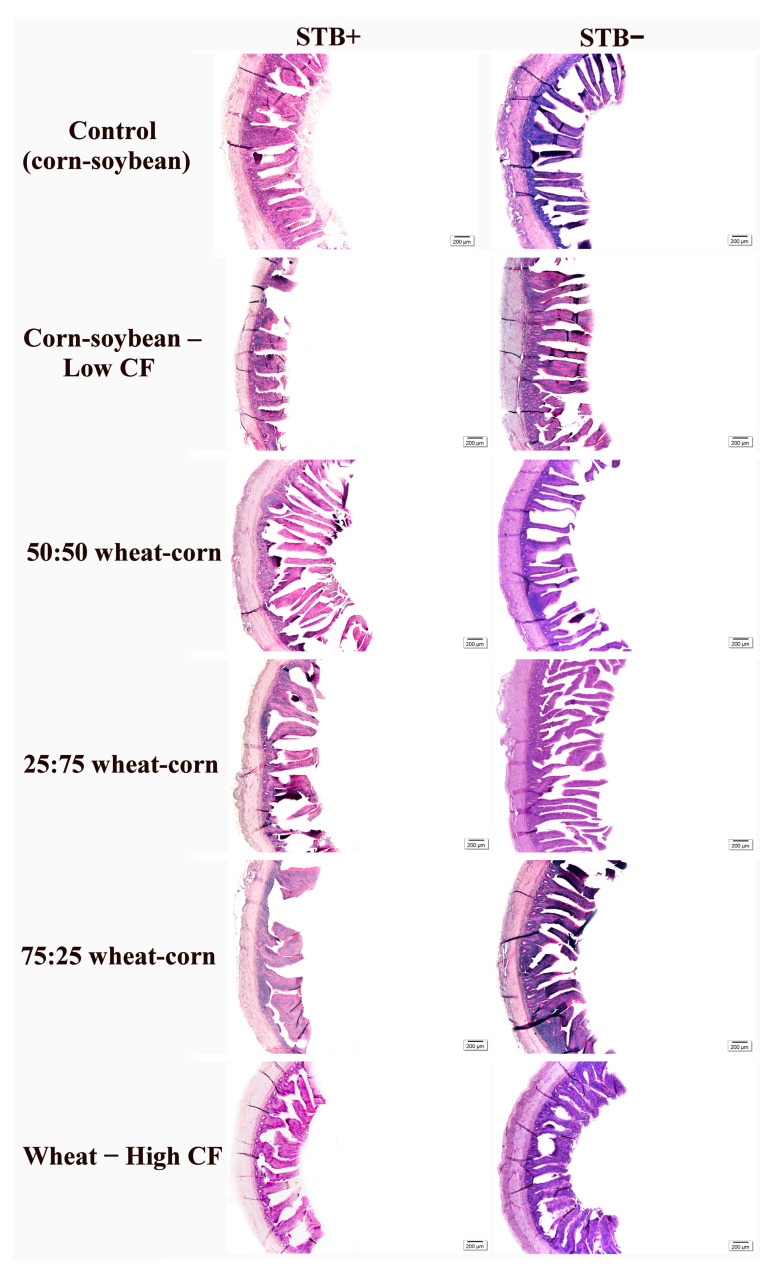
Photomicrographs of the ileum in laying hens, supplemented with dietary fiber levels and STB supplementation. Control (corn–soybean); Corn–soybean—Low CF; 50:50 wheat–corn; 25:75 wheat–corn; 75:25 wheat–corn e Wheat—High CF. Staining: PAS. Scale bars: 200 µm.

Interactions between STB supplementation and dietary fiber levels were observed for duodenum, jejunum and ileum morphology in laying hens (*p* < 0.0001) (Table 4). The main effect of STB supplementation promoted wider villi (*p* < 0.0001) and a greater absorptive area in the jejunum (*p* = 0.0063). In the ileum, hens not receiving STB supplementation exhibited taller villi (*p* = 0.0092) and deeper crypts (*p* = 0.0054). The Control (commercial diet), Corn–soybean, and Corn–soybean diets also influenced ileal morphology, promoting wider villi (*p* = 0.0025) and a greater absorptive area. Representative images of the intestinal morphology under different dietary fiber levels and STB supplementation are shown in Figure 1, Figure 2 and Figure 3.

The specific interactions between dietary fiber levels and STB supplementation. In the duodenum, hens fed the Corn–soybean diet with STB exhibited wider villi (*p* = 0.0106). Conversely, the 25:75 wheat–corn diet with STB reduced villus width, while the 50:50 wheat–corn diet without STB resulted in shallower crypts (*p* = 0.0011). Additionally, the Corn–soybean diet with STB decreased the villus-to-crypt ratio (*p* = 0.0058), whereas the Corn–soybean diet with STB increased the absorptive area (*p* = 0.0086). In the ileum, hens receiving the 50:50 wheat–corn diet with STB presented narrower villi (*p* = 0.0011), a lower villus-to-crypt ratio (*p* = 0.0058), and a reduced absorptive area (*p* = 0.0086).

## 4. Discussion

The aim of the current study was to evaluate the effect of different dietary high-fiber levels, with or without STB supplementation, on the productive performance, egg quality, and intestinal morphology of laying hens. According to our results, the data suggest that the metabolism and physiological response of the birds were more strongly modulated by the amount and type of dietary fiber than by the addition of the supplement, although positive effects from the combination of high fiber levels and STB were identified.

Diets with intermediate wheat–corn ratios (25:75 and 75:25) resulted in better productive performance—reflected by higher egg production and egg mass, as well as improved feed conversion—possibly due to greater digestive and energetic efficiency resulting from a more balanced intestinal fermentation. Dietary fiber plays a multifactorial role in the metabolism of poultry [20]. At moderate levels, especially when composed of fermentable fractions such as non-starch polysaccharides (NSPs), it stimulates cecal microbiota activity, promoting fermentation and the consequent production of short-chain fatty acids (SCFAs), including acetate, propionate, and butyrate [1,8,14]. These metabolites are absorbed by the intestinal epithelium and used as energy sources by enterocytes, contributing to mucosal integrity and nutrient absorption efficiency [5]. Walugembe et al. [1] observed that moderate fiber inclusion in the diet increased cecal SCFA concentrations and positively influenced the cecal microbiota in broilers and laying hens aged 1 to 21 days, which aligns with our findings of improved body weight variation (BWV) and nutritional efficiency in hens fed diets containing 25:75 and 75:25 wheat–corn ratios.

Conversely, excessive fiber inclusion, as observed in the 50:50 wheat–corn diet, increased feed intake without proportional gains in egg production. This behavior reflects a compensatory physiological response in which birds increase feed consumption to compensate for the lower energy density of the diet [21]. Similar findings were reported by Singh and Kim [14], who noted that high-fiber diets can reduce nutrient digestibility and impair performance when the fermentable substrate exceeds optimal levels. However, it is suggested that excessive insoluble fiber without the use of STB may accelerate intestinal transit and reduce digestion time, compromising nutrient absorption and resulting in lower feed efficiency and greater body weight loss.

Diets with high NSP concentrations tend to increase intestinal content viscosity, hindering the diffusion of digestive enzymes and substrates and consequently reducing starch and protein hydrolysis [5]. This lower digestibility may lead to reduced glucose and amino acid availability in the intestinal lumen, affecting energy metabolism and protein synthesis—processes essential for yolk and albumen formation and, therefore, for egg quality. This allows us to infer that, in the groups with the best performance (Control, corn–soybean, and 75:25 wheat–corn), the more balanced energy availability favored efficient oxidation of carbohydrates and lipids, ensuring adequate energy for egg production and basal metabolism while reducing body reserve mobilization.

The results we observed in egg production and quality are certainly directly associated with the intestinal morphology outcomes. Diets with moderate fiber levels were also associated with improved intestinal morphology, featuring taller and wider villi and higher villus-to-crypt ratios, which increase absorptive surface area and enhance digestive efficiency [22]. A more developed intestinal environment favors both nutrient absorption and microbiota stability. Conversely, excessive fiber can cause mild mucosal irritation, stimulating mucin production and epithelial turnover—processes that increase energy expenditure and reduce productive efficiency [14].

Although STB supplementation did not show an isolated significant effect, it may exert modulatory functions under specific dietary conditions. According to Kouzounis et al. [23], β-1,4-endo-xylanase and xylo-oligosaccharides promote partial hydrolysis of NSPs, reducing intestinal viscosity and releasing fermentable oligosaccharides that serve as substrates for beneficial microorganisms such as Lactobacillus and Bifidobacterium. Thus, it is likely that the positive effects of STB occur synergistically with specific fiber levels, optimizing microbial fermentation and nutrient absorption rather than acting independently.

Egg quality traits—including yolk color, shell thickness, and specific gravity—were influenced by both fiber levels and STB supplementation. STB supplementation promotes partial hydrolysis of NSPs, reducing intestinal viscosity and releasing fermentable oligosaccharides. These compounds stimulate the growth of beneficial microorganisms, such as Lactobacillus and Bifidobacterium, which in turn promote SCFA production and enhance the absorption efficiency of essential minerals like calcium and phosphorus—key elements for eggshell formation [23]. Thus, the greater specific gravity and shell thickness observed in STB-supplemented diets may reflect improved mineral bioavailability mediated by changes in the microbiota and intestinal fermentation. Corn–soybean-based diets produced heavier eggs with darker yolks, a result associated with the presence of dietary pigments (carotenoids and xanthophylls) and improved nutrient absorption, which enhance yolk pigmentation and albumen quality [12,13]. STB supplementation improved eggshell characteristics, particularly specific gravity, likely due to partial hydrolysis of arabinoxylans and increased mineral bioavailability in the intestine, as suggested by Nguyen et al. [13] and Akhtar et al. [1]. These findings reinforce that the effects of STB are diet-dependent, being influenced by fiber source, particle size, and solubility [12]. According to our results, diets with balanced fiber levels (especially 25:75 and 75:25 wheat–corn) associated with STB promote a more efficient intestinal environment—from both biochemical and microbiological perspectives—which positively affects nutrient absorption efficiency and, consequently, yolk pigmentation, albumen integrity, and eggshell quality.

Our results also showed a significant interaction between dietary fiber levels and STB supplementation on intestinal morphology, particularly in the duodenum and ileum, supporting its prebiotic role in stimulating beneficial microbial fermentation and SCFA production [12,24]. STB exerted a main effect by increasing villus width and the absorptive area of the jejunum, indicating improved intestinal mucosal integrity and functionality. In contrast, non-supplemented hens exhibited taller villi in the ileum but deeper crypts—a morphological pattern that may reflect higher epithelial cell turnover, possibly in response to digestive or fermentative challenges caused by high-fiber diets [25]. This finding may also have a mechanistic explanation, suggesting that excessive fiber hydrolysis can disrupt the microbiota and reduce epithelial support mediated by SCFAs, thereby affecting intestinal morphology [14].

The duodenum and jejunum are the primary regions responsible for nutrient digestion and absorption. The increased villus width promoted by STB in these regions is likely physiologically associated with higher expression of glucose and amino acid transporters, which optimizes absorption and reduces fecal energy losses. This effect translates into better metabolic nutrient utilization and potentially greater productive efficiency, even indirectly.

Our findings demonstrate that intestinal morphology is strongly influenced by the interaction between dietary fiber and enzymatic supplementation. Diets with balanced wheat–corn ratios (25:75 and 75:25) combined with STB tend to promote a more stable and functional intestinal environment, reflected in wider villi and greater absorptive areas. Conversely, excessive soluble fiber (50:50 wheat–corn) may cause adverse structural changes even with STB use, suggesting a physiological limit to the benefits of NSP fermentation. Thus, we hypothesize that optimal combinations of fermentable fiber and STB may act synergistically to enhance productivity, intestinal health, and egg quality in laying hens.

## 5. Conclusions

High dietary fiber levels (specifically, the 25:75 and 75:25 wheat–corn ratios) improved laying hens’ performance, egg quality, and intestinal morphology, and supplementation with 0.01% stimbiotic further enhanced these effects, highlighting it as an effective strategy to optimize production and gut health in commercial laying hens.

Further studies are needed to better elucidate the use of STB and different levels and sources of fiber for laying hens.

## Figures and Tables

**Table 1:** Feedstuff and chemical composition (g/kg) of diets for Bovans White laying hens.

Items	Control (Commercial Diet)	Corn–Soybean	75:25 Wheat–Corn	50:50 Wheat–Corn	25:75 Wheat–Corn	Wheat–Soybean
Wheat	169.0	0.0	449.2	299.5	149.7	598.9
Wheat bran	0.0	0.0	128.4	85.6	42.8	171.2
Corn	535.3	565.1	145.3	285.2	425.1	5.4
Corn gluten meal	0.0	221.0	55.3	110.5	165.8	0.0
Soybean meal	195.1	111.3	111.5	111.4	111.4	111.5
Soybean oil	1.3	3.7	8.7	7.0	5.3	10.3
Coarse limestone	48.0	47.7	43.6	43.3	43.0	48.5
Fine limestone	32.0	31.9	37.0	37.0	37.0	32.3
Dicalcium phosphate	8.7	6.9	7.4	7.3	7.1	7.6
Salt	2.5	1.0	1.6	1.4	1.2	1.8
Sodium bicarbonate	1.0	1.0	1.6	1.4	1.2	1.7
L-Lysine HCl, 780 g/kg	1.0	2.6	2.4	2.5	2.6	2.3
DL-Methionine, 999 g/kg	1.8	1.8	2.0	1.9	1.8	2.0
L-Threonine, 985 g/kg	0.0	0.2	0.6	0.4	0.3	0.7
L-Tryptophan, 980 g/kg	0.0	0.6	0.2	0.3	0.5	0.0
L-Valine, 990 g/kg	0.0	0.0	0.4	0.2	0.1	0.5
L-isoleucina	0.1	0.9	0.8	0.9	0.9	0.8
Choline chloride, 600 g/kg	2.3	2.3	2.3	2.3	2.3	2.3
Vitamin premix and trace mineral ^1^	2.0	2.0	2.0	2.0	2.0	2.0
Stimbiotic (STB) ^2^	0.0	0.0	0.0	0.0	0.0	0.0
Total	1000	1000	1000	1000	1000	1000
Calculated nutrient content, g/kg						
ME kcal/kg	2830	2670	2670	2670	2670	2670
Crude protein	163	164	164	164	164	165
Met + Cys dig	7.0	7.0	7.0	7.0	7.0	7.0
Lys dig	7.4	7.4	7.4	7.4	7.4	7.4
Tre dig	5.3	5.3	5.3	5.3	5.3	5.3
Tryp dig	1.8	1.8	1.8	1.8	1.8	1.8
Val dig	6.0	6.0	6.0	6.0	6.0	6.0
Ca	334	334	334	334	334	334
Available P	3.9	3.9	3.9	3.9	3.9	3.9
Na	1.7	1.7	1.7	1.7	1.7	1.7
CF	23.4	37.3	29.9	32.4	34.9	27.4
Neutral detergent fiber	101	161	132	142	151	122
Acid detergent fiber	43.2	58.2	58.4	58.3	58.3	58.4

^1^ Vitamin premix provided per kg of product: vitamin A, 9637 UI; vitamin D3, 2409 UI; vitamin E, 36.1 UI; vitamin K3, 1.93 mg; vitamin B1, 2.59 mg; vitamin B12, 0.016 mg; vitamin B6, 3.61 mg; vitamin B5, 12.95 mg; vitamin B3, 39.0 mg; vitamin B9, 0.90 mg; biotin, 0.09 mg; Trace mineral provided per kg of product: Mn, 64.20 mg; Zn, 59.63 mg; Fe, 45.85 mg; Cu, 9.14 mg; I, 0.927 mg; Se, 0.275 mg. ^2^ Signis, β-1,4-endo-xylanase and xylooligosaccharides, AB Vista, Marlborough, UK.

**Table 2:** Influence of dietary fiber levels and STB supplementation on the performance of laying hens.

Diets		FI (g/Layer/Day)	EP (%)	EM (g)	FCR-EM (kg/kg)	FCR-DZ (kg/Dozen)	BWV (g)
Means for main effect of STB							
STB	+	117.55	94.46	56.41	2.10	1.50	6.81
	−	117.27	95.05	56.87	2.08	1.48	8.08
Means for main effect of fiber levels							
Control (commercial diets)		115.65 b	96.10 a	57.92 a	2.01 d	1.45 c	11.07 a
Corn–soybean		117.13 ab	91.72 c	54.24 c	2.17 a	1.54 a	6.00 b
50:50 wheat–corn		118.46 a	94.98 ab	56.68 ab	2.10 bc	1.50 ab	7.44 ab
25:75 wheat–corn		117.6 ab	93.62 bc	55.52 bc	2.12 ab	1.51 ab	6.16 b
75:25 wheat–corn		118.30 a	95.97 a	57.66 a	2.07 bcd	1.48 bc	6.13 b
Wheat–soybean		117.49 ab	96.15 a	57.83 a	2.05 cd	1.47 bc	7.88 ab
Pooled SEM		0.216	0.243	0.174	0.007	0.005	0.478
*p*-Value	STB	0.5042	0.1637	0.0908	0.0743	0.0697	0.1600
	Fiber levels	0.0029	<0.0001	<0.0001	<0.0001	<0.0001	0.0152
	Interaction	0.4499	0.4077	0.8681	0.4592	0.2274	0.2084

The values are means of 100 layers per treatment (N = 100 layers per treatment); STB, stimbiotic; FI, Feed intake; EP, Egg production; EM, Egg mass; FCR-EM, Feed Conversion Ratio per egg mass; FCR-DZ, feed conversion dozen eggs; BWV, Body weight variation; a,b,c,d, Means within a column, in the same group, followed by different lowercase letters differ significantly according to the F-test (STB) and Tukey’s test (fiber levels) at *p* < 0.05.

**Table 3:** Influence of dietary fiber levels and STB supplementation on the egg quality in laying hens.

Diets		Egg Weight (g)	Yolk (%)	Albumen (%)	Eggshell (%)	Yolk Color	Haugh Unit	Shell t Hickness (mm)	Specific Gravity (g/cm^3^)
Means for main effect of STB									
STB	+	59.63	26.01	63.84	10.15	4.08	94.10 a	0.411	1.18
	−	59.90	25.88	63.94	10.17	4.17	93.70 b	0.411	1.16
Means for main effect of fiber levels									
Control (commercial diets)		60.35 a	26.05	63.81	10.17	4.85	93.14 d	0.412	1.159
Corn–soybean		58.82 c	26.39	63.41	10.18	5.03	94.59 a	0.409	1.185
50:50 wheat–corn		59.66 ab	25.44	64.43	10.11	4.22	94.50 ab	0.410	1.173
25:75 wheat–corn		59.31 bc	25.76	63.97	10.27	4.76	94.10 abcd	0.411	1.166
75:25 wheat–corn		60.08 a	26.05	63.81	10.10	3.05	93.46 cd	0.410	1.170
Wheat–soybean		60.29 a	26.96	63.92	10.10	2.84	93.77 bcd	0.413	1.166
Pooled SEM		0.088	0.106	0.197	0.021	0.104	0.093	0.001	0.003
*p*-Value	STB	0.1097	0.5632	0.6779	0.5797	0.4853	0.0222	0.8505	<0.0001
	Fiber levels	<0.0001	0.2586	0.3190	0.1419	<0.0001	<0.0001	0.0198	<0.0001
	Interaction	0.6820	0.3484	0.4067	0.1310	<0.0001	0.0674	<0.0001	<0.0001
Interaction									
STB	Fiber levels								
+	Control (commercial diets)	60.12	25.82	64.00	10.25	4.770 Aa	93.54	0.419 Aa	1.154 Ac
	Corn–soybean	60.23	26.06	63.83	10.09	4.731 Aa	93.86	0.404 Bc	1.212 Aa
	50:50 wheat–corn	59.83	25.37	64.65	9.99	3.704 Bc	94.33	0.407 Ab	1.185 Ab
	25:75 wheat–corn	59.05	25.75	63.92	10.33	5.476 Aa	94.38	0.411 Ab	1.179 Ab
	75:25 wheat–corn	59.92	26.04	63.86	10.1	3.270 Abc	94.00	0.406 Ab	1.179 Ab
	Wheat–soybean	58.62	27.03	62.79	10.15	2.542 Ac	94.51	0.417 Aab	1.165 Aac
−	Control (commercial diets)	60.58	26.29	63.61	10.11	4.936 Aab	92.73	0.405 Bb	1.164 Aa
	Corn–soybean	60.34	25.86	64.02	10.12	5.337 Aa	93.68	0.414 Aa	1.159 Ba
	50:50 wheat–corn	59.5	25.51	64.21	10.24	4.729 Aab	94.65	0.413 Aa	1.162 Ba
	25:75 wheat–corn	59.56	25.77	64.02	10.23	4.038 Bbc	93.81	0.411 Aab	1.155 Ba
	75:25 wheat–corn	60.24	26.07	63.77	10.11	2.822 Ac	92.92	0.410 Aab	1.162 Aa
	Wheat–soybean	59.02	25.76	64.03	10.23	3.131 Ac	94.66	0.411 Aab	1.168 Aa

The values are means of 30 eggs per treatment in each evaluation period (N = 30 eggs per treatment); STB, stimbiotic; A, B, uppercase letters compare the inclusion or not of STB within columns; a, b, c, d, lowercase letters compare dietary fiber levels within rows; significantly dif-ferent (*p* < 0.05) according to Tukey’s test.

**Table 4:** Influence of dietary fiber levels and STB supplementation on the villus width (VW, μm), villus height (VH, μm), crypt depth (CD, μm), villus height-to-crypt depth ratio (VH:CD), and villus absorptive area (AA, µm^2^) of duodenum, jejunum and ileum morphology in laying hens.

Diets		Duodenum					Jejunum					Ileum				
		VW	VH	CD	VH:CD	AA	VW	VH	CD	VH:CD	AA	VW	VH	CD	VH:CD	AA
STB	+	227.9	1475.8	130.9	11.4	335,961.4	174.4 a	1241	123.3	10.2	219,140 a	120.2	689.0 b	82.1	8.4	85,329
	−	222	1472	128.9	11.5	327,658.7	147.9 b	1170.2	118.7	9.9	175,594.7 b	122.9	755.2 a	91	8.4	94,820.9
Main effects	Control	236.8	1450.1	124.5	11.7	343,478.4	156.3	1113.2	116.5	9.5	172,678.4	125.8	759.6	90.4	8.5	96,595.7
	Corn–soybean	236.3	1440.7	129.2	11.2	335,886.3	163.4	1251.4	125.3	10	211,014.4	130.9	749.1	89.1	8.5	100,142.9
	50:50 wheat–corn	224.9	1409.9	130.4	10.9	319,300.4	157.9	1176.8	125.2	9.5	186,341.5	119.1	714.3	88.3	8.2	91,060.6
	25:75 wheat–corn	220.4	1546.2	134.6	11.6	342,153	168.7	1211.4	120.1	10.1	209,486.9	107.2	688.8	82.4	8.4	75,438.8
	75:25 wheat–corn	213.2	1444.7	129.1	11.3	308,635	156.3	1230.1	119	10.4	194,397.3	118.5	746.3	87	8.5	84,733.4
	Wheat–soybean	223.1	1447.5	130.8	11.2	324,053.7	146.1	1114.9	115.8	9.6	163,737.4	126.1	679.9	86.6	7.9	92,480.3
	Pooled SEM	0.052	0.053	0.037	0.035	0.073	0.086	0.08	0.053	0.042	0.133	0.066	0.07	0.061	0.038	0.114
*p*-Value	STB	0.2749	0.5182	0.9377	0.4139	0.6161	0.0001	0.6331	0.7308	0.4119	0.0063	0.5508	0.0092	0.0054	0.464	0.0641
	Fiber levels	0.0924	0.2949	0.1923	0.2771	0.2774	0.5587	0.3727	0.2715	0.0592	0.2404	0.0025	0.1829	0.5037	0.2029	0.0156
	Interaction	0.0106	0.2262	0.0011	0.0058	0.0086	0.0742	0.848	0.1193	0.6487	0.1753	0.0011	0.2348	0.0058	0.1507	0.0086
Interaction + STB	Control	235.6 Aab	1403.1	120.2 Aa	11.7 Aa	331,621.9 Aab	172.5	1125.4	112.4	10	190,428.5	133.5 Aa	751	92.5 Aa	8.2	101,858.8 Aa
	Corn–soybean	258.0 Aa	1407.9	133.8 Ab	10.5 Bb	355,183.2 Aa	169.3	1297.9	127.1	10.3	229,518.3	124.2 Aa	720.1	84.0 Aab	8.7	91,952.6 Aa
	50:50 wheat–corn	223.5 Aab	1411.1	137.6 Ab	11.2 Aab	339,579.5 Aab	169.7	1286.5	125	10.5	219,187.7	94.0 Bb	744.9	73.6 Bb	8.7	57,953.4 Bb
	25:75 wheat–corn	208.0 Ab	1520.6	128.1 Aab	11.2 Aab	296,733.4 Ab	140.8	1081.9	110.9	9.8	149,898.2	121.5 Aa	591.4	84.6 Aab	8.1	93,264.8 Aa
	75:25 wheat–corn	211.6 Aab	1504.4	131.0 Ab	11.6 Aa	320,082.3 Aab	192.8	1204.5	121.2	10.1	233,044.7	116.6 Aa	668.5	79.7 Aab	8.4	80,661.3 Aab
	Wheat–soybean	239.7 Aab	1436	128.3 Aab	11.3 Aab	346,183.9 Aab	172	1207.1	131	9.4	208,783	134.5 Aa	696.4	88.2 Aab	7.9	95,276.0 Aa
Interaction − STB	Control	238.1 Aa	1497.2	128.9 Aab	11.7 Aa	355,334.9 Aa	140.2	1101	120.7	9.2	154,928.4	118.1 Aa	768.3	88.3 Aa	9	91,332.6 Aa
	Corn–soybean	214.6 Aa	1473.6	124.6 Ab	11.8 Aa	316,589.3 Aab	157.5	1205	123.6	9.8	192,510.5	137.6 Aa	778.2	94.2 Aa	8.4	108,333.2 Aa
	50:50 wheat–corn	222.8 Aa	1478.4	124.0 Bb	11.2 Aab	308,527.9 Ab	142.9	1173.7	113.1	10.4	169,606.9	120.5 Aa	747.8	99.7 Aa	8.4	92,924.2 Aa
	25:75 wheat–corn	218.4 Aa	1374.5	130.1 Aab	11.4 Aab	320,536.7 Aab	151.4	1147.9	120.8	9.6	177,576.7	116.7 Aa	768.4	89.4 Aa	7.8	88,856.3 Aa
	75:25 wheat–corn	229.1 Aa	1588.2	138.2 Aa	11.6 Aa	364,223.6 Aa	144.7	1218.4	119.1	10.2	185,929.2	120.4 Aa	709.1	85.1 Aa	8.4	88,805.6 Aa
	Wheat–soybean	210.0 Aa	1384	132.4 Aab	10.5 Ab	292,417.0 Bb	143.9	1146.6	119.4	9.7	163,900	117.6 Aa	732.2	88.5 Aa	8.6	89,684.7 Aa

The values are means of 90 measurements per variable per treatment (N = 90 measurements per treatment); STB, stimbiotic; A, B, uppercase letters compare the inclusion or not of STB within columns; a, b, lowercase letters compare dietary fiber levels within rows; significantly different (*p* < 0.05) according to Tukey’s test.

## Data Availability

Dataset available on request from the authors.
